# Medical Pathology and Therapeutics, and Practical Medicine

**Published:** 1854-08

**Authors:** 


MEDICAL PATHOLOGY AND THERAPEUTICS, AND PRACTICAL MEDICINE. *Pyrosis, its Causes, Pathology and Treatment.*—Dr. GEO. BUDD, in an interesting lecture on Pyrosis, observes it is most probable that the disorder in countries in which it is endemic, is mainly owing to the influence of climate and to the diet of the poor not being sufficiently varied, and consisting too much of coarse and innutritious farinaceous food.

But if such be the main causes of the disorder, there are, doubtless, various other conditions that assist in bringing it on. Most of these may be classed under two heads:

1. Excessive labor, insufficient clothing, loss of blood, and all other conditions that tend to exhaust the body.

2. Pregnancy, constipation, anxiety, and other conditions that tend to disorder the functions of the stomach.

We have already seen that waterbrash occasionally occurs in a high degree in the wealthy classes, especially in women, where it cannot be ascribed to any peculiarity in diet, and seems to be owing solely to such conditions as these.

Pyrosis, then, considered with reference to its exciting causes, is of two kinds:

1. That which has been termed by some writers *symptomatic* pyrosis, which is brought on (without any peculiarity in diet) by pregnancy or some other condition that disturbs the functions of the stomach.

2. That which has been termed, in contradistinction to the former, *idiopathic* pyrosis, which prevails chiefly among the agricultural poor in rural districts, and which seems, in most cases, to be mainly owing to defective diet.

Many conditions conspire to render the disorder much more frequent in women than in men. Women are much more frequently in states of debility from the nature of their constitutions, and from their having in suckling and in excessive or unnatural uterine discharges, causes of exhaustion from which men are exempt; they have also more excitable nervous systems, and, in consequence, the functions of the stomach in them are more apt to be deranged by mental influences and by disease in other parts of the body; and among the lower classes, they have generally a less nutritious diet, since the men, in order to support their more laborious work, take or have accorded to them a larger quantity of animal food and of malt liquors than is consumed by the weaker sex.

In the treatment of waterbrash, our first endeavor should, of course, be to remove the conditions that may seem to have brought it on or to maintain it.

If the disorder should seem to be caused mainly by a diet not sufficiently nutritious, or consisting too much of farinaceous substances, the most effectual remedy will be a wholesome nourishing diet, containing a proper quantity of animal food *in its most digestible form.* Little permanent benefit can, indeed, be expected from medicine unless the diet is improved.

If the disorder should seem to have been induced, or to be kept up, wholly or in part, by fatigue, it is very essential that the patient should rest; if by constipation, that this condition should be removed by purgatives, such as aloes or colocynth, that do not offend the stomach.

After these points have been attended to, much further good may be done by medicines.

The medicines that have been found most useful in pyrosis are:

1. Medicines which have an astringent action on the coats of the stomach. Among these may be classed bismuth, lime-water, and the vegetable astringents—kino, catecu, krameria, logwood.

2. Sedatives, especially opium and the salts of morphia, which probably also tend to restrain undue secretion by the mucous membrane, but which are chiefly of use in allaying the gastralgia that attends pyrosis.

Medicines from these two classes may often be combined with advantage. Five grains of bismuth with a twelfth of a grain of the muriate of morphia, or five grains of the compound kino powder, or an efficient dose of catechu, krameria, or logwood, with opium, may be given two or three times a day.

3. Some other medicines have obtained repute in pyrosis which cannot be classed with the preceding. They have most of them an astringent action on the coats of the stomach, but act, directly or indirectly, on the nervous system as well.

The chief of these are, nitrate of silver, which may be given in pills, in doses, of half a grain, three times a day; nux vomica, which may also be given in pill, in the dose of from three to five grains, three times a day; quinia; and the mineral acids.

Some of the medicines I have mentioned have been popular remedies for pyrosis in districts in which the malady has prevailed.

It is stated that nux vomica is a popular remedy among the Laplanders, to whom it was recommended by Linnæus, and that lime-water was some years ago a popular remedy among the rural population of North Wales.

4. The disorder is often connected with anæmia; and steel is of great service, both in removing it and in preventing its recurrence.

The medicines of which I have had most experience in disorders of this class, and which are probably as efficacious as any, are bismuth, with morphia; krameria, and logwood, with opium; and steel.—*Med. Times and Gaz.*

